# Reappraisal of the malignant risk of thyroid nodules with isolated macrocalcifications in thyroidology

**DOI:** 10.1016/j.clinsp.2025.100794

**Published:** 2025-10-14

**Authors:** Ilker Sengul, Demet Sengul

**Affiliations:** a Thyroidology Unit, Giresun University Faculty of Medicine, Giresun, Turkey; b Division of Endocrine Surgery, Giresun University Faculty of Medicine, Giresun, Turkey; c Department of General Surgery, Giresun University Faculty of Medicine, Giresun, Turkey; d Department of Pathology, Giresun University Faculty of Medicine, Giresun, Turkey

*Dear Editor*,

The debate is still ongoing regarding the sonographic features of the delicate endocrine gland and their implications for prospective tumor biology in the state of affairs in thyroidology.^1^, ^2^, ^3^, ^4^, ^5^, ^6^, ^7^ We do pen these lines concerning the review article entitled “Malignant risk of thyroid nodules with isolated macrocalcifications – A study based on surgery results”, set forth within the esteemed journal *Clinics*, volume 80.^1^ Yang and colleagues^1^ do reveal and address a crucial and burgeoning area of research in the controversial topic of the malignancy risk associated with Isolated Macrocalcifications (IMC) in thyroid nodules, a significant point of discussion as calcifications are common on ultrasound. The authors provide valuable data based on surgical outcomes, which they suggest offers more reliable information than studies based solely on Fine-Needle Aspiration (FNA) or Core-Needle Biopsy (CNB), particularly given the potential for false-negative results in nodules with macrocalcifications. The finding that focal disruption of the anterior margin of IMC was significantly associated with malignancy is particularly noteworthy and aligns with some previous research highlighting the importance of irregular margins. However, the study presents several limitations. Being a retrospective study from a single center and limited to patients who underwent surgery with confirmed histopathology might introduce a notable selection bias. As such, the sample size of 46 IMC nodules, derived from 3680 consecutive thyroidectomy patients, is relatively small, particularly when nodules are subdivided by size. Furthermore, this limited sample size and retrospective design may explain some of the study's findings that appear to contradict established guidelines or other research.^1^ Of note is that the study exhibited no significant difference in malignancy risk among IMC nodules of different size groups (maximum diameter <10 mm, 10–14 mm, and >15 mm),^1^, ^2^, ^3^, ^4^ which contrasts with guidelines like K-TIRADS, which suggest FNA for IMC nodules larger than 10 mm, and some studies proposing 15 mm as a better cutoff. The authors suggest their small sample size might account for this discrepancy. Similarly, the study revealed no significant difference in Lymph Node Metastasis (LNM) rates among the different size groups, which differs from studies reporting higher LNM rates in larger tumors, although the optimal cutoff for predicting LNM remains debated. The authors again note that the retrospective design limits conclusions regarding tumor size as a prognostic factor in IMC nodules. Moreover, while the reported overall malignancy risk of 30.43 % for IMC nodules based on surgical outcomes is higher than that reported in some FNA/CNB-based studies, this variability could also be a function of the specific population studied and the inherent differences in diagnostic methods. In essence, the study provides valuable surgical outcome data supporting the association between focal disruption of IMC and malignancy risk. Nevertheless, its retrospective, single-center nature and limited sample size, particularly acknowledged by the authors, mean that the findings regarding nodule size and risk stratification should be interpreted with caution. As the authors correctly state, to this end, large-scale prospective studies are required to definitively establish the malignancy risk of IMC nodules and refine the cutoff values for further investigation.

The 2015 American Thyroid Association (ATA) Management Guidelines for Adult Patients with Thyroid Nodules and Differentiated Thyroid Cancer stated that multivariable analyses confirm a higher probability of cancer for nodules with either microlobulated margins or microcalcifications than for hypoechoic solid nodules lacking these features.^5^^,^^6^ In addition, it was reported that macrocalcifications within a nodule, if combined with microcalcifications, confer the same malignancy risk as microcalcifications alone.^5^, ^6^, ^7^ Nevertheless, the presence of isolated intranodular macrocalcification is not consistently associated with thyroid cancer.^5^^,^^10^ In retaliation, a nodule possessing interrupted peripheral calcifications, with a soft tissue rim outside the calcification, is highly likely to be malignant, which might demonstrate tumor invasion in disrupted calcification.^5^^,^^8^^,^^9^ This issue merits further investigation.^1^ We thank Yang et al.^1^ for their valuable study in *Clinics*, on the malignant risk of thyroid nodules with isolated macrocalcifications for thyroidologists. Bene diagnoscitur, bene curatur.

## Abbreviation

IMC, Isolated Macrocalcifications; FNA, Fine-Needle Aspiration; CNB, Core-Needle Biopsy; LNM, Lymph Node Metastasis.

## Authors’ contributions

Ilker Sengul: Conceptualization; formal analysis; ınvestigation; methodology; project administration; resources; software; validation; visualization; writing-original draft; writing-review& editing.

Demet Sengul: Conceptualization; formal analysis; ınvestigation; methodology; project administration; resources; software; supervision; validation; visualization; writing-original draft; writing-review& editing.

All authors have read and approved the final and published version of the manuscript.

## Declaration of competing interest

The authors declare no conflicts of interest.
